# Morphological Diversity of Turtle Hyoid Apparatus is Linked to Feeding Behavior

**DOI:** 10.1093/iob/obae014

**Published:** 2024-05-02

**Authors:** G Jorgewich-Cohen, I Werneburg, M Jobbins, G S Ferreira, M D Taylor, D Bastiaans, M R Sánchez-Villagra

**Affiliations:** Department of Paleontology, University of Zurich, 8006 Zurich, Switzerland; Senckenberg Centre for Human Evolution and Palaeoenvironment an der Universität Tübingen, Tübingen, Germany; Fachbereich Geowissenshcaten dr Universität Tübingen, 72074 Tübingen, Germany; Department of Paleontology, University of Zurich, 8006 Zurich, Switzerland; Senckenberg Centre for Human Evolution and Palaeoenvironment an der Universität Tübingen, Tübingen, Germany; Fachbereich Geowissenshcaten dr Universität Tübingen, 72074 Tübingen, Germany; School of Biological Sciences, The University of Western Australia, Perth, WA 6009, Australia; The UWA Oceans Institute, The University of Western Australia, Perth, WA 6009, Australia; Department of Paleontology, University of Zurich, 8006 Zurich, Switzerland; Department of Paleontology, University of Zurich, 8006 Zurich, Switzerland

## Abstract

The hyoid apparatus of tetrapods is highly diverse in its morphology. It plays an important role in feeding, breathing, sound production, and various other behaviors. Among turtles, the diversity of the hyoid apparatus has been recurrently linked to their habitat. The ossification of the hyoid corpus is often the main trait used in correlations with “niche” occupancy, an ossified corpus being associated with aquatic environments and a cartilaginous corpus with terrestrial life. Most studies conducted so far have focused on species belonging to Testudinoidea, the clade that occupies the biggest diversity of habitats (i.e., terrestrial, semi-terrestrial, and aquatic animals), while other turtle lineages have been largely understudied. We assessed the adult anatomy of the hyoid apparatus of 92 turtle species from all “families”, together with ossification sequences from embryological series of 11 species, some described for the first time here. Using nearly 40 different discrete anatomical characters, we discuss the evolutionary patterns and the biological significance of morphological transformations in the turtle hyoid elements. Morphological changes are strongly associated to feeding modes, with several instances of convergent evolution within and outside the Testudines clade, and are not as strongly connected to habitat as previously thought. Some of the hyoid character states we describe are diagnostic of specific turtle clades, thus providing phylogenetically relevant information.

## Synopsis (Portuguese) - Resumo

O aparelho hióide dos tetrápodes é altamente diversificado em sua morfologia. Ele desempenha um papel importante na alimentação, respiração, produção de som e vários outros comportamentos. Entre as tartarugas, a diversidade do aparelho hióide tem sido recorrentemente associada ao habitat ocupado pela espécie. A ossificação do corpo hióide é frequentemente a principal característica utilizada nas correlações com ocupação de “nicho”, com um corpus ossificado associado a ambientes aquáticos e um corpus cartilaginoso a ambientes terrestres. A maioria dos estudos realizados até agora focaram em espécies pertencentes a Testudinoidea, o clado que ocupa o maior diversidade de habitats (ou seja, animais terrestres, semi-terrestres e aquáticos), enquanto outras linhagens de tartarugas têm sido pouco estudadas. Avaliamos a anatomia adulta do aparelho hióide de 92 espécies de tartarugas de todas as “famílias”, juntamente com sequência de ossificação de séries embriológicas de onze espécies, algumas descritas pela primeira vez aqui. Usando quase 40 caracteres anatômicos discretos diferentes, discutimos os padrões evolutivos e o significado biológico das transformações morfológicas nos elementos hióides da tartaruga. Alterações morfológicas estão fortemente associadas aos modos de alimentação, com vários exemplos de evolução convergente dentro e fora do clado Testudines, e não estão tão fortemente ligados ao habitat como se pensava anteriormente. Alguns dos estados de caráter que descrevemos são diagnósticos de clados específicos de tartarugas, fornecendo assim informações filogeneticamente relevantes.

## Synopsis (Spanish) - Resumen

El aparato hyoideo de los tetrápodos es muy diverso en su morfología. El mismo desempeña un papel importante en la alimentación, la respiración, la producción de sonidos y otras funciones. En las tortugas, la diversidad del aparato hyoideo se ha relacionado con el hábitat. La osificación del cuerpo hyoideo es a menudo el rasgo principal utilizado en correlaciones con la ocupación de “nichos”; un cuerpo osificado se asocia con ambientes acuáticos y un cuerpo cartilaginoso con la vida terrestre. La mayoría de los estudios realizados se han centrado en especies pertenecientes a Testudinoidea, el clado que ocupa la mayor diversidad de hábitats (terrestre, semiterrestre y acuático), mientras que otros linajes de tortugas han sido poco estudiados. Evaluamos la anatomía adulta del aparato hyoideo de 92 especies de tortugas de todas las “familias”, junto con secuencias de osificación de series embriológicas de once especies, algunas descritas por primera vez aquí. Utilizando casi 40 caracteres anatómicos discretos diferentes, discutimos los patrones evolutivos y el significado biológico de las

transformaciones morfológicas en los elementos hyoideos de las tortugas. Los cambios morfológicos están asociados con los modos de alimentación, con varios casos de convergencia dentro y fuera del clado Testudines; la asociación con el hábitat no es tan robusta como se ha hipotetizado. Algunos de los estados de caracteres que describimos son diagnósticos de clados de tortugas específicos, proporcionando así información filogenéticamente relevante.

## Synopsis (German)

Der Zungenbeinapparat der Tetrapoden ist morphologisch sehr vielfältig. Er spielt eine wichtige Rolle bei der Nahrungsaufnahme, der Atmung, der Tonerzeugung und verschiedenen anderen Verhaltensweisen. Bei Schildkröten wurde die Vielfalt des Zungenbeinapparats immer wieder mit ihrem Lebensraum in Verbindung gebracht. Die Verknöcherung des Zungenbeins ist häufig das Hauptmerkmal, um ökologische Nischen-Zugehörigkeiten zu besprechen, wobei ein verknöchertes Hyoid-Corpus mit aquatischen Lebensräumen und ein knorpeliges mit terrestrischer Lebensweise in Verbindung gebracht wird. Die meisten Studien konzentrierten sich bisher auf Arten, die zu den Testudinoidea gehören, der Gruppe mit der größten Vielfalt an Lebensräumen (d. h. terrestrische, semi-terrestrische und aquatische Tiere), während andere Schildkrötengruppen bisher kaum untersucht wurden. In der vorliegenden Studie haben wir die adulte Anatomie des Zungenbeinapparats von 92 Arten aus allen Schildkröten-„Familien“untersucht. Hinzu kommen Verknöcherungssequenzen aus embryologischen Serien von elf Arten, von denen einige hier zum ersten Mal beschrieben werden. Anhand von fast 40 diskreten anatomischen Merkmalen diskutieren wir die evolutionären Muster und die biologische Bedeutung der morphologischen Veränderungen des Hyoid-Apparats. Sie stehen in engem Zusammenhang mit der Art der Nahrungsaufnahme, wobei es mehrere Fälle konvergenter Evolution innerhalb und außerhalb der Schildkröten gibt. Einige der von uns beschriebenen Hyoid-Merkmale sind diagnostisch für bestimmte Taxa und liefern somit phylogenetisch relevante Informationen.

## Introduction

The hyoid apparatus (or hypobranchial apparatus) present in modern tetrapods is partly homologous to the visceral skeleton of fishes (Romer and Parsons 1970) and plays a fundamental role in the lives of these animals. Many functions of the hyoid apparatus, including its role in feeding, breathing, and sound production are analogous across tetrapods (Tanner and Avery 1982). However apomorphic innovations are observed, like the unique moving tongue of the alligator snapping turtle, *Macrochelys temminckii*, used to attract prey; and the dewlap extensions of anoles and other lizards (Spindel et al. 1987; Bels 1990; Ord et al. 2015).

Within the different utilities of the hyoid apparatus, feeding appears to represent the strongest source of selection for its morphological diversity (Schwenk 2000; Wainwright et al. 2015). Among fish, capturing food through suction (i.e., rapid decrease of intraoral pressure by expanding the buco-pharyngeal chamber) is a widespread behavior (Van Damme and Aerts 1997; Wainwright et al. 2015). During the process of colonization of land, however, a wide array of unique morphological features evolved in tetrapods in association with specific feeding habits (Schwenk 2000).

Among tetrapods, few animal lineages have developed suction as a form of underwater feeding, a characteristic that evolved in association with secondary events of recolonization of water (Wainwright et al. 2015; Barrionuevo 2016; Molnar et al. 2022). Among animals that have acquired this behavior are several lineages of frogs, salamanders, (although in some amphibian lineages suction feeding is a plesiomorphic trait), and turtles (Deban and Wake 2000; Nishikawa 2000; Deban 2003; Barrionuevo 2016; Lautenschlager et al. 2018); and some birds and mammals (e.g., flamingos and whales; Wainwright et al. 2015). In amphibians, this ability appeared recurrently in over a dozen lineages that, while not sharing a direct common ancestor (Barrionuevo 2016), display correlated convergent morphological features, such as a highly ossified hyoid apparatus and loss or reduction of the tongue (Nishikawa 2000; Deban 2003).

Among turtles, some of the morphological characters of the hyoid apparatus are hypothesized to be associated with habitat preference, with terrestrial clades (i.e., tortoises and some members of the Geoemydidae and Emydidae) showing a cartilaginous structure, while aquatic species tend to have highly ossified elements (Richter et al. 2007; Lemell et al. 2019). Regardless of each species’ habitat preferences, feeding strategies vary greatly in Testudines (Herrel et al. 2002). Considering the prior studies on other tetrapod groups and specific turtle lineages (e.g., Van Damme and Aerts 1997; Natchev et al. 2015), one can expect these feeding habits to greatly influence the morphology of the hyoid apparatus.

Although some work has attempted to reassess the morphological diversity in the hyoid apparatus of some turtle clades (e.g., Siebenrock 1898, 1913; Schumacher 1973; Haiduk 1979; Richter et al. 2007), the lack of a systematic and comprehensive study including all clades makes any conjectures about the drivers of diversity speculative. There is therefore a need to evaluate the morphological diversity of the turtle hyoid apparatus across a broader range of species from all Testudines (crown-group turtles). It is also important, in this context, to examine embryologic and ontogenetic patterns, considering different degrees of ossification of the hyoid apparatus—as they have been reported to change during the life of several turtle species (Tanner and Avery 1982; Richter et al. 2007).

With the aim of understanding patterns of embryological development of the hyoid apparatus, in a phylogenetic framework, we started by describing ontogenetic changes in the ossification of the hyoid apparatus of *Pelodiscus sinensis* WIEGMANN 1835. We then discuss the heterochronic changes in comparison to previously studied species. Finally, we analyze the diversity present in the structures that compose the hyoid apparatus from 116 specimens representing 92 species from all major taxa (“families”) based on 39 morphological characters. We discuss our morphological findings in the context of the current knowledge about the ecological traits and phylogeny of turtles.

## Methods

We analyzed the embryological developmental sequence of the hyoid apparatus of *P. sinensis* (Sánchez-Villagra et al. 2009), arranging the specimens based on days of incubation instead of using developmental “stages” to avoid subjective categorizations (sensu Werneburg 2009). We refer the reader to Tokita and Kuratani (2001) for further information on *P. sinensis*. Photographs were made with a digital camera (Leica DFC420 C) attached to a stereomicroscope at a resolution of 2592 × 1944 pixels.

Information about the ontogeny of ossification of the hyoid apparatus in other turtle species was extracted from museum specimens (*n* = 4) and from the literature (*n* = 5: Siebenrock 1913; Haiduk 1979; Kuratani 1999; Sheil and Greenbaum 2005; Richter et al. 2007; Bona and Alcade 2009; Mohamad et al. 2023). The only species in which the onset of hyoid apparatus ossification during embryogenesis has been described to a similar level of detail to that of *P. sinensis* in this study are *Caretta caretta* (Kuratani 1999), *Chelydra serpentina* (Sheil and Greenbaum 2005), and *Phrynops hillarii* (Bona and Alcade 2009). For these species comparison was possible for both embryonic and post-hatchling development of the hyoid apparatus. For the remaining museum specimens described for the first time here (*Rafetus euphraticus, Staurotypus salvinii, Chelonoidis nigra*, and *Podocnemis expansa*), as well as those of *Trachemys scripta* (Haiduk 1979), *Cuora galhimifrores* (Richter et al. 2007), and *Mauremys capsicar* (Siebenrock 1913) only details and comparison of post-hatching ossification were possible. We did not include information from additional studies where descriptions of hyoid ossification were less detailed such as in Sánchez-Villagra et al. (2009)—*P. sinensis*, and Werneburg et al. (2009)—*Emydura subglobosa*.

We calculated temporal shifts in the ossification sequence units that constitute the hyoid apparatus using an event pair matrix (a method that allows encoding of event sequences to be analyzed as phylogenetic characters; Smith 2001) and used the ranked values to plot a principal component analysis (PCA) to enable visualization of species distribution in association with vectors that represent the most influential traits in the ossification sequences.

We also aimed to compare the hyoid apparatus of adult turtles across the widest possible phylogenetic coverage by compiling information from museum specimens, dissections, and µCT-scans (Tables 1 and 2, respectively). We mostly used scanned specimens from Ferreira et al. (2020, 2022). In addition, we µCT-scanned ten specimens with a Nikon XT H 320 micro-tomography scanner at the 3D imaging lab of the University of Tübingen, Germany. Specimens were prepared before scanning using a 2% iodine (I2) solution diluted in ethanol (70%) for contrast. We injected the solution into the specimens once a week, and they were submerged in the solution for 45 days. The segmentation and 3D-model reconstructions were performed using Mimics v. 21.0 (Materialise, Leuven, Belgium) and Avizo software (Thermo Fischer Scientific, Waltham, MA, USA).

**Table 1:** List of examined specimens from skeletal collections and dissections

**Species**	**Collection**	**Number**	**Family**
*Emys orbigularis*	PIMUZ	A/III796	Emydidae
*Chrysemys picta*	NMW	2216	Emydidae
*Clemmys guttata*	NMW	2237	Emydidae
*Deirochelys reticularia*	NMW	39,106	Emydidae
*Graptemys geographica*	NMW	1717	Emydidae
*Graptemys pulchra*	NMW	41,014	Emydidae
*Terrapene carolina*	NMW	215	Emydidae
*Terrapene ornata*	NMW	216	Emydidae
*Trachemys dorbigni*	NMW	41,352	Emydidae
*Trachemys ornata*	NMW	2228	Emydidae
*Platysternon megacephalum*	NMW	41,567_10	Platysternidae
*Chelonoidis carbonaria*	NMW	38267	Testudinidae
*Chelonoidis denticulatus*	NMW	223	Testudinidae
*Chelonoidis nigra microphyes*	NMW	221	Testudinidae
*Geochelone pardalis*	NMW	38,230	Testudinidae
*Kinixys belliana*	NMW	1885	Testudinidae
*Manouria impressa*	NMW	39,100	Testudinidae
*Psammobates oculifer*	NMW	2123	Testudinidae
*Stigmochelys pardalis*	NMW	2309	Testudinidae
*Testudo kleinmanni*	NMW	1936	Testudinidae
*Testudo marginata*	NMW	217	Testudinidae
*Cuora amboinensis*	NMW	214	Geoemydidae
*Cuora mouhotii*	NMW	37,197	Geoemydidae
*Cyclemys dentata*	NMW	2236	Geoemydidae
*Geoemyda spengleri*	NMW	37,279	Geoemydidae
*Heosemys depressa*	NMW	1758	Geoemydidae
*Mauremys mutica*	NMW	39,101	Geoemydidae
*Mauremys rivulata*	NMW	2203	Geoemydidae
*Melanochelys tricarinata*	NMW	39,134	Geoemydidae
*Morenia ocellata*	NMW	39,668–1	Geoemydidae
*Orlitia borneensis*	NMW	2318	Geoemydidae
*Pangshura sylhetensis*	NMW	40,009	Geoemydidae
*Pangshura tentoria*	NMW	39,014_1	Geoemydidae
*Rhinoclemmys pulcherrima*	NMW	2217	Geoemydidae
*Rhinoclemmys rubida*	NMW	1779	Geoemydidae
*Kinosternon integrum*	NMW	2239	Kinosternidae
*Kinosternon leucostomum*	NMW	2218	Kinosternidae
*Kinosternon scorpioides*	NMW	2224	Kinosternidae
*Staurotypus salvinii*	NMW	2227	Kinosternidae
*Sthernotherus carinatus*	NMW	39,875–1	Kinosternidae
*Sthernotherus odoratus*	NMW	2235	Kinosternidae
*Dermatemys mawii*	NMW	30,836	Dermatemydidae
*Chelydra serpentina*	NMW	208	Chelydridae
*Macrochelys temminckii*	NMW	41,339	Chelydridae
*Caretta caretta*	NMW	19,898	Cheloniidae
*Chelonia mydas*	NMW	1860	Cheloniidae
*Eretmochelys imbricata*	NMW	3210	Cheloniidae
*Dermochelys coriacea*	NMW	30,159	Dermochelyidae
*Carettochelys insculpta*	NMW	41,541	Carettochelyidae
*Amyda cartilaginea*	NMW	231	Trionychidae
*Apalone mutica*	PIMUZ	A/III795	Trionychidae
*Apalone spinifera*	NMW	2243	Trionychidae
*Chitra indica*	NMW	238	Trionychidae
*Cyclanorbis senegalensis*	NMW	229	Trionychidae
*Dogania subplana*	NMW	233	Trionychidae
*Lissemys punctata*	NMW	2186	Trionychidae
*Nilssonia cf. gangetica*	NMW	230	Trionychidae
*Pelochelys cantorii*	NMW	237	Trionychidae
*Pelodiscus sinensis*	NMW	234	Trionychidae
*Rafetus euphraticus*	NMW	2317	Trionychidae
*Trionyx triunguis*	NMW	232	Trionychidae
*Acantochelys pellidipectoris*	NMW	40,161_1	Chelidae
*Acantochelys spixii*	NMW	40,728	Chelidae
*Chelodina oblonga*	NMW	227	Chelidae
*Chelus fimbriata*	PIMUZ	A/III957	Chelidae
*Emydura macquarii*	NMW	2215	Chelidae
*Hydromedusa tectifera*	NMW	40,284	Chelidae
*Phrynops geoffroanus*	NMW	228	Chelidae
*Erymnochelys madagascariensis*	NMW	2313	Podocnemididae
*Peltocephalus dumerilianus*	INPA	–	Podocnemididae
*Podocnemis expansa*	PIMUZ	A/III951	Podocnemididae
*Pelomedusa galeata*	NMW	2222	Pelomedusidae
*Pelomedusa subrufa*	NMW	41,408_1	Pelomedusidae

**Table 2:** Species subjected to µCT-scan in the present study

**“Family”**	**Species**	**Collection**	**Number**	**Data reference**
Carettochelyidae	*Carettochelys insculpta*	USNM	327,960	Rollot et al. (2023)
Chelidae	*Chelus fimbriata*	ZMB Herpetologie	9088	Werneburg and Ferreira (2024)
Chelidae	*Chelus fimbriata* (hatchling)	GPIT	GPIT-PV-108,377	Werneburg and Ferreira (2024)
Chelidae	*Emydura subglobosa*	GPIT	GPIT-PV-122,906	Ferreira and Werneburg (2021), Werneburg and Ferreira (2024)
Chelidae	*Platemys platycephala*	SMNS	uncatalogued	Ferreira and Werneburg (2021), Werneburg and Ferreira (2024)
Chelidae	*Phrynops hillarii*	IW	1140	No permission to share data (see Werneburg et al. 2015a suppl. mat. for specimen information)
Cheloniidae	*Caretta caretta*	GPIT	GPIT-PV-108,375	Ferreira and Werneburg (2021), Werneburg and Ferreira (2024)
Chelydridae	*Chelydra serpentina*	R	14,442	Ferreira and Werneburg (2021), Werneburg and Ferreira (2024)
Dermochelyidae	*Dermochelys coriacea*	GPIT	GPIT-PV-108,376	Ferreira and Werneburg (2021), Werneburg and Ferreira (2024)
Emydidae	*Emys orbicularis*	SMNS	11,390	Ferreira and Werneburg (2021), Werneburg and Ferreira (2024)
Emydidae	*Graptemys pseudogeographica*	SMNS	3702	Ferreira and Werneburg (2021), Werneburg and Ferreira (2024)
Emydidae	*Terrapene carolina*	SMNS	7434	Ferreira and Werneburg (2021), Werneburg and Ferreira (2024)
Emydidae	*Trachemys scripta elegans*	IW	1131	No permission to share data (see Werneburg et al. 2015a suppl. mat. for specimen information)
Geoemydidae	*Cuora amboinensis*	SMNS	4867_2	Ferreira and Werneburg (2021), Werneburg and Ferreira (2024)
Geoemydidae	*Mauremys caspica*	SMNS	5354	Werneburg and Ferreira (2024)
Geoemydidae	*Heosemys grandis*	GPIT	GPIT-PV-108,380	Werneburg and Ferreira (2024)
Kinosternidae	*Kinosternon subrubrum*	R	10,089	Ferreira and Werneburg (2021), Werneburg and Ferreira (2024)
Pelomedusidae	*Pelusios niger*	SMNS	4625	Ferreira and Werneburg (2021), Werneburg and Ferreira (2024)
Platysternidae	*Platysternon megacephalum*	R	12,559	Ferreira and Werneburg (2021), Werneburg and Ferreira (2024)
Podocnemididae	*Podocnemis erythrocephala*	SMNS	6063_1	Ferreira and Werneburg (2021), Werneburg and Ferreira (2024)
Testudinidae	*Testudo hermanni*	SMNS	11,424_3	Werneburg and Ferreira (2024)
Trionychidae	*Apalone spinifera aspera*	R	12,970	Ferreira and Werneburg (2021), Werneburg and Ferreira (2024)

IW, internal data-ID of Ingmar Werneburg.

## Taxonomic sampling

Dissections and observation of previously prepared skeletons were conducted in several institutions, including the Department of Paleontology of the University of Zurich (PIMUZ), the National Institute of Amazonian Research (INPA), the Natural History Museum of Vienna (NMW), the Western Australian Museum, the Smithsonian National Museum of Natural History (USNM), and the Palaeontological Collection of the University of Tübingen (GPIT).

The hyoid apparatus was segmented from CT-scans of 23 specimens belonging to 22 species (Table 2).

## Phylogenetic relationships, character optimization, and terminology

**Fig. 1:**
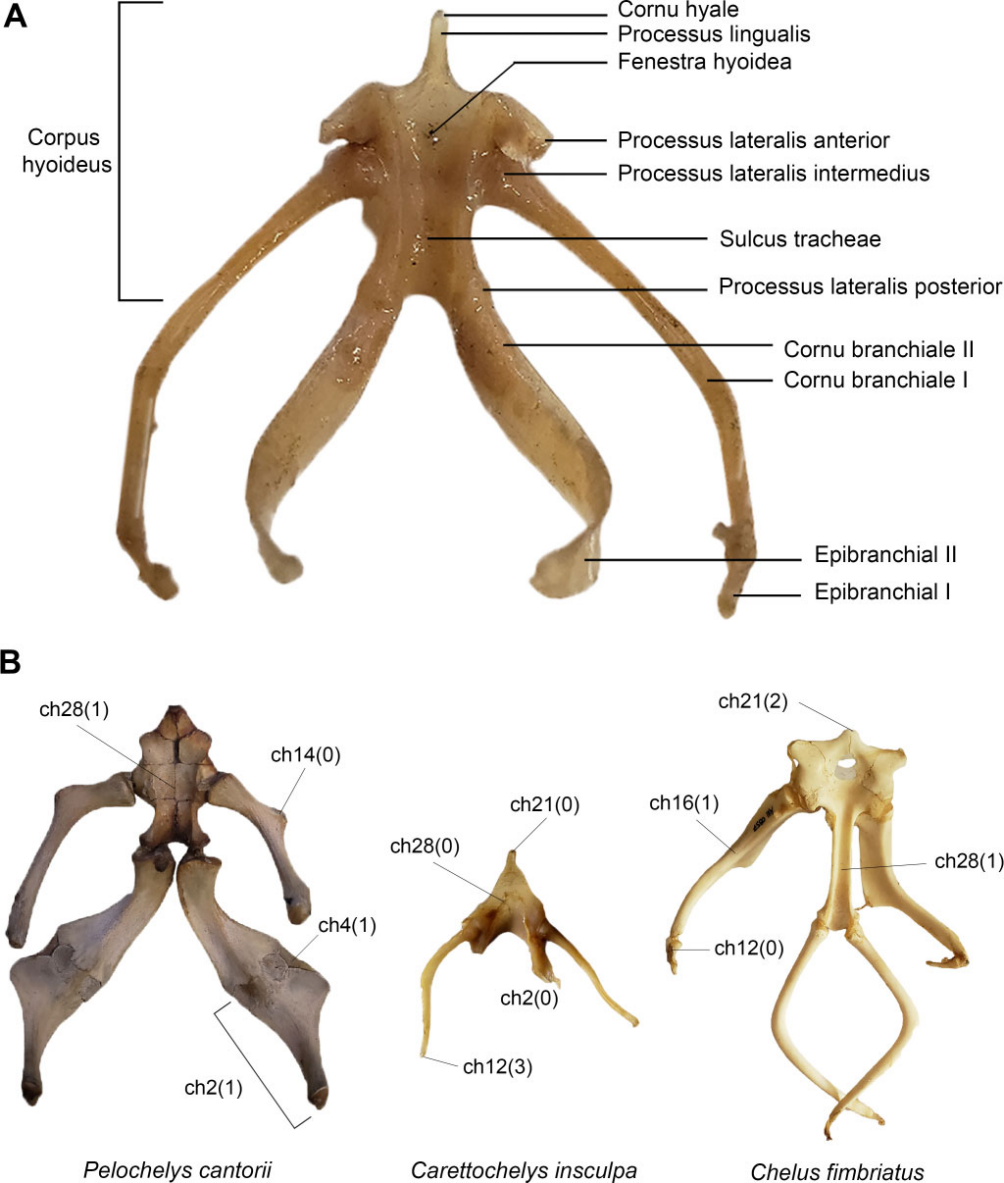
Nomenclature for hyoid structures and examples of character states (see Table 3 for a complete list).

We used Mesquite version 3.51 (Maddison and Maddison 2018) to create the character matrix (Table 3, Fig. 1, Supplemental Material 1) and TNT version 1.5 (Goloboff and Catalano 2016) to optimize the character states to a modified version of the phylogenetic hypothesis proposed by Pereira et al. (2017). Although there is a more recent phylogenetic hypothesis available (Thomson et al. 2021), their differences are only minor within-group relations and Pereira et al. (2017) hypothesis has the greatest overlap with our dataset. We also provide reconstructions of ancestral states of each character, including ambiguous optimizations (Supplemental Material 2).

**Table 3:** List of characters used in the present study, based on adult specimens

**EPIBRANCHIAL II**
1. Epibranchial II: (0) absent; (1) cartilage; (2) bone
2. Epibranchial II, number of bony parts: (0) only one element; (1) two elements; (2) several unstandardised, asymmetrical elements (possibly different numbers in each side)
3. Epibranchial II, proportions (all together): (0) wider than long; (1) longer than wide; (2) as long as wide
4. Presence of cartilage attachment edge that links CBII to a bony element of Epibranchial II that is not in direct contact with CBII: (0) absence of edge; (1) presence of edge
5. Position of the attachment edge with respect to the longitudinal axis: (0) internal; (1) external
6. Attachment grooves at the end of Epibranchials II: (0) absent; (1) present
**CORNU BRANCHIALE II**
7. Cornu branchiale II, ossification: (0) cartilaginous; (1) ossified
8. Cornu branchiale II, relative shape: (0) proximal end wider than distal end; (1) distal end is wider than proximal end; (2) proximal and distal end have the same width
9. Cornu branchiale II, shape: (0) tubular; (1) flattened; (2) tubular in the distal end and flattened in the proximal end; (3) flattened in the distal end and tubular in the proximal end
10. Cornu branchiale II, frill: (0) without frill (or expansion); (1) with frill
**EPIBRANCHIAL I**
11. Epibranchial I: (0) absent; (1) cartilaginous; (2) ossified
12. Epibranchials I, shape: (0) hook-like; (1) cylindrical; (2) oval and flat; (3) conical
**CORNU BRANCHIALE I**
13. Cornu branchiale I, relative shape: (0) proximal end wider than distal end; (1) distal end wider than proximal end; (2) proximal and distal end have the same width
14. Cornu branchiale I, attachment site for musculature at the medial, curved portion (“elbow's”): (0) presence of attachment hinges; (1) hinges not evident
15. Cornu branchiale I, attachment site for musculature at the proximal portion (“wrist's”): (0) present; (1) absent
16. Cornu branchiale I, frill: (0) absent; (1) present
17. Cornu branchiale I, frill location: (0) at the “elbow”; (1) at the “wrist”; (2) between “elbow and “wrist”
18. Cornu branchiale I, length: (0) longer than cornu branchiale II; (1) shorter than cornu branchiale II
**PROCESSUS LINGUALIS**
19. Processus lingualis, ossification: (0) cartilaginous; (1) partially ossified; (2) fully ossified
20. Processus lingualis, attachment: (0) separation from corpus hyoideus distinguishable; (1) separation from corpus hyoideus indistinguishable
21. Processus lingualis, shape: (0) appendage; (1) isosceles; (2) equilateral
**FENESTRA HYOIDEA**
22. Fenestra hyoidea: (0) absent; (1) present
23. Fenestra hyoidea, gap: (0) visible boundaries (occluded by synchondroses); (1) open
24. Fenestra hyoidea, relation to hyoid body: (0) enclosed by the corpus hyoideus; (1) anterior part is open (also includes cases where it is enclosed in the distal end by cartilage only while all the rest is ossified)
25. Fenestra hyoidea, fusion: (0) one; (1) two
26. Fenestra hyoidea, relative size to space between processi lateralis intermedius: (0) thinner than sulcus tracheae's width; (1) wider than ST's width; (2) as wide
**SULCUS TRACHEAE**
27. Sulcus tracheae, ossification: (0) cartilaginous; (1) ossified
28. Sulcus tracheae, bony parts: (0) only one bone that is fused to corpus hyoideus without clear separation; (1) only one bone that is not fused with the rest of the corpus hyoideus; (2) four bony elements (from processus lateralis posterior to intermedius) that are not fused to the rest of the corpus hyoideus.
29. Sulcus tracheae, relative width: (0) anterior and posterior part present same width; (1) anterior wider; (2) posterior wider
30. Sulcus tracheae, relative shape: (0) longer than wider; (1) as long as wide; (2) wider than long
**CORPUS HYOIDEUS**
31. Corpus hyoideus, ossification: (0) cartilaginous; (1) partially ossified with islands; (2) ossified throughout length
32. Corpus hyoideus, anterior end structure when ossified: (0) subdivided into separate bony elements; (1) fused
33. Corpus hyoideus, length between processi: (0) space between processi lateralis intermedius wider than space between processi lateralis posterior; (1) space between processi lateralis intermedius as wide as space between processi lateralis posterior; (2) Space between processi lateralis posterior wider than space between processi lateralis intermedius
34. Corpus hyoideus, position of processi: (0) process lateralis intermedius closer to processus lateralis anterior; (1) processus lateralis intermedius closer to processus lateralis posterior; (2) equal distances
35. Corpus hyoideus (including sulcus tracheae), shape: (0) spoon like; (1) polygon; (2) triangular
36. Corpus hyoideus size: (0) at least twice as long (from processus lateralis posterior to anterior) as wide (measured at processus lateralis intermedius); (1) longer than wide, but shorter than twice the width; (2) as long as wide, (3) wider than long
**CORNU HYALE**
37. Cornu hyale: (0) absent; (1) cartilaginous; (2) ossified
38. Cornu hyale, shape: (0) claw-like with curvature; (1) stalklike; (2) conical; (3) triangular and flat; (4) elongated oval
39. Cornu hyale, attachment: (0) presence of suture, clearly separating from processus lateralis anterior; (1) absence of suture, elements are fused

Anatomical nomenclature mostly follows Sheil and Greenbaum (2005), as they presented the most complete and consistent terminology. A brief overview of nomenclature variation present in the literature is available in Supplemental Material 3.

In respect to the highly debated issue of character coding—such as the “no tail, red tail, blue tail” problem (see, for instance, Maddison 1993; Hawkins et al. 1997; Brazeau 2011; Evers and Benson 2019)—crucial information to define strong homology hypotheses are currently not available. We therefore decided for an instrumental approach in order to avoid resolution problems in subsequent analyses caused by null character states resultant from correlated characters in specimens that lack specific structures (Maddison 1993).

## Results

**Fig. 2:**
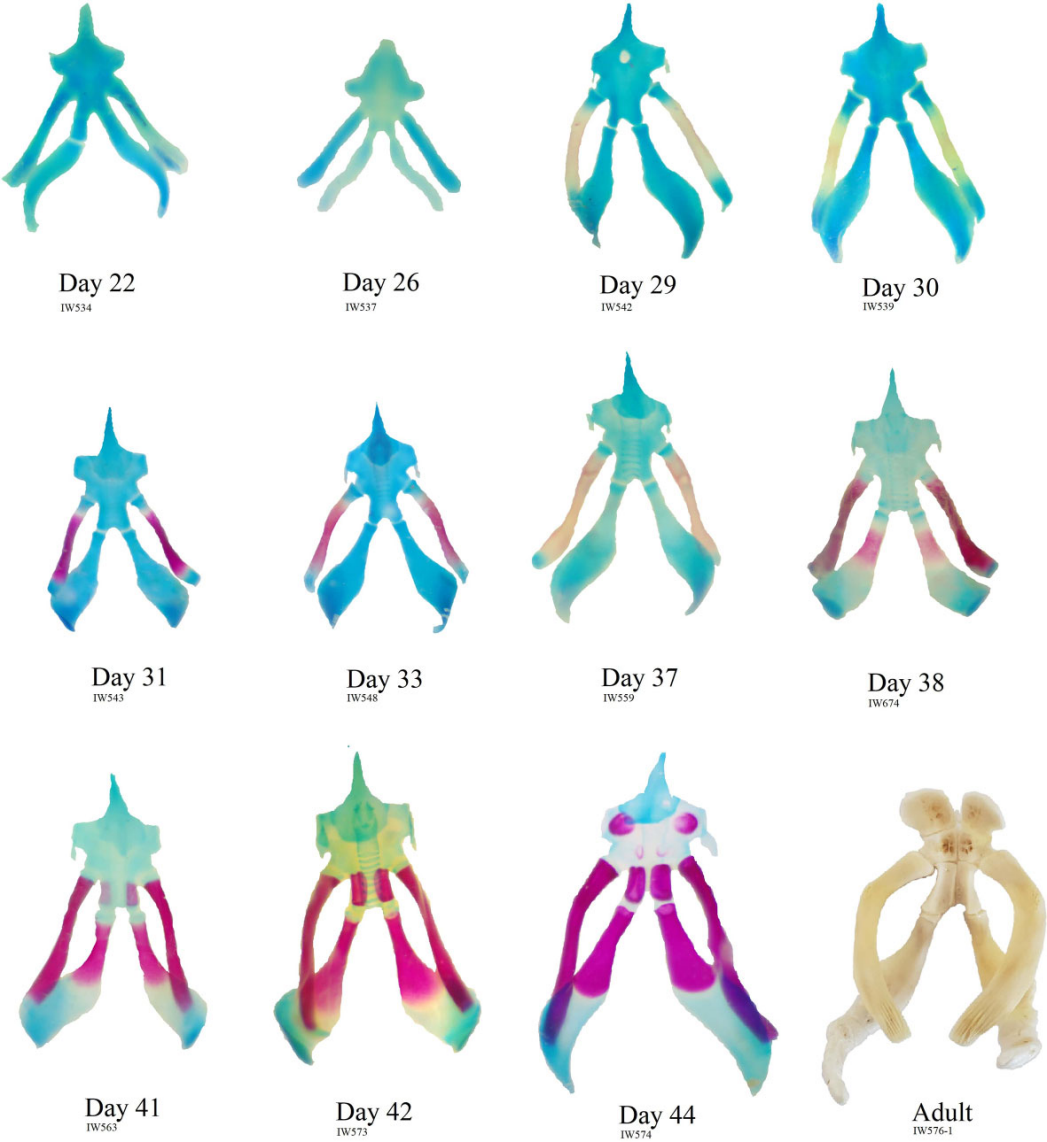
Embryological series of a *Pelodiscus sinensis* hyoid apparatus. Level of ossification is represented by color with catilagenous structures being stained blue/green and bone red/purple structures. An example of a fully ossified, unstained, adult hyoid apparatus is provided for visual comparison. Image not to scale.

The embryological series of *P. sinensis* allows clear visualization of the ossification patterns during its development (Fig. 2).

*Pelodiscus sinensis*, day 22–26

Until the 26th day of incubation, no ossification center can be observed. Projections of the cornu hyale are absent, and the proximal ends of the cornu branchiale are not distinguishable from the epibranchials.

*Pelodiscus sinensis*, days 29–33

The central portion of the cornu branchiale I is the first element to ossify, occurring by day 29. The aperture of the fenestra hyoidea and the projections of the cornu hyale are also first visible at this developmental stage. By day 31, the cornu branchiale retains considerable amounts of Alizarin Red stain, and the ossification center expands toward the extremities—especially the posterior end, being easily differentiated from the epibranchial I. By day 33 the fenestra hyoidea is completely closed.

*Pelodiscus sinensis*, days 37–38

On the 37th day of incubation, the first evidence of ossification can be found in the central portion of the cornu branchiale II. At day 38th this ossification center is already expanded and retains substantial amounts of Alizarin Red.

*Pelodiscus sinensis*, days 41–42

The cornu branchiale I is highly ossified by the 41st day, with a small remnant of the epibranchial I. At this stage, the posterior end of the corpus hyoideus exhibits a paired center of ossification that will later become the sulcus tracheae. The ossification center of the cornu branchiale II has expanded considerably in both directions. On the 42nd day, the ossification of cornu branchiale II has greatly expanded toward the epibranchials.

*Pelodiscus sinensis*, day 44

The Alizarin Red stains great amounts of all ossification centers described in previous embryonic stages. Cornu branchiale I and II are fully ossified, and there are no visible traces of the epibranchials I. The paired centers of ossification in the posterior end of the corpus hyoideus are now expanded and are in contact. Two new paired centers of ossification can be visualized in the corpus hyoideus at this stage—one in the anterior end and one in the medial portion, between the other two-paired centers. The degree of ossification of the most anterior paired center, based on size and amount of stain, indicates that its appearance precedes the medial paired center.

*Pelodiscus sinensis*, adult

In the adult specimen, all structures are fully ossified, except for the processus lingualis, and the cornu hyale—the latter being absent or reminiscent. The epibranchial I is completely absent, but attachment hinges are present in the posterior end of the cornu hyale I. The epibranchial II is ossified with random, unpaired ossification centers that can vary from 5 to 12 (based on the 3 specimens available to us). The corpus hyoideus is subdivided into 3 pairs of bones. The anterior pair is the only one in which the bones are not in direct contact, creating an opening to which the posterior end of the cartilaginous processus lingualis is attached. This opening does not seem to represent the fenestra hyoidea, as the fenestra is a transient structure in the embryological development of this species, only present between the 29th and 30th days of development, closing in the following stages. It is more likely just an attachment site of the processus lingualis.

**Fig. 3:**
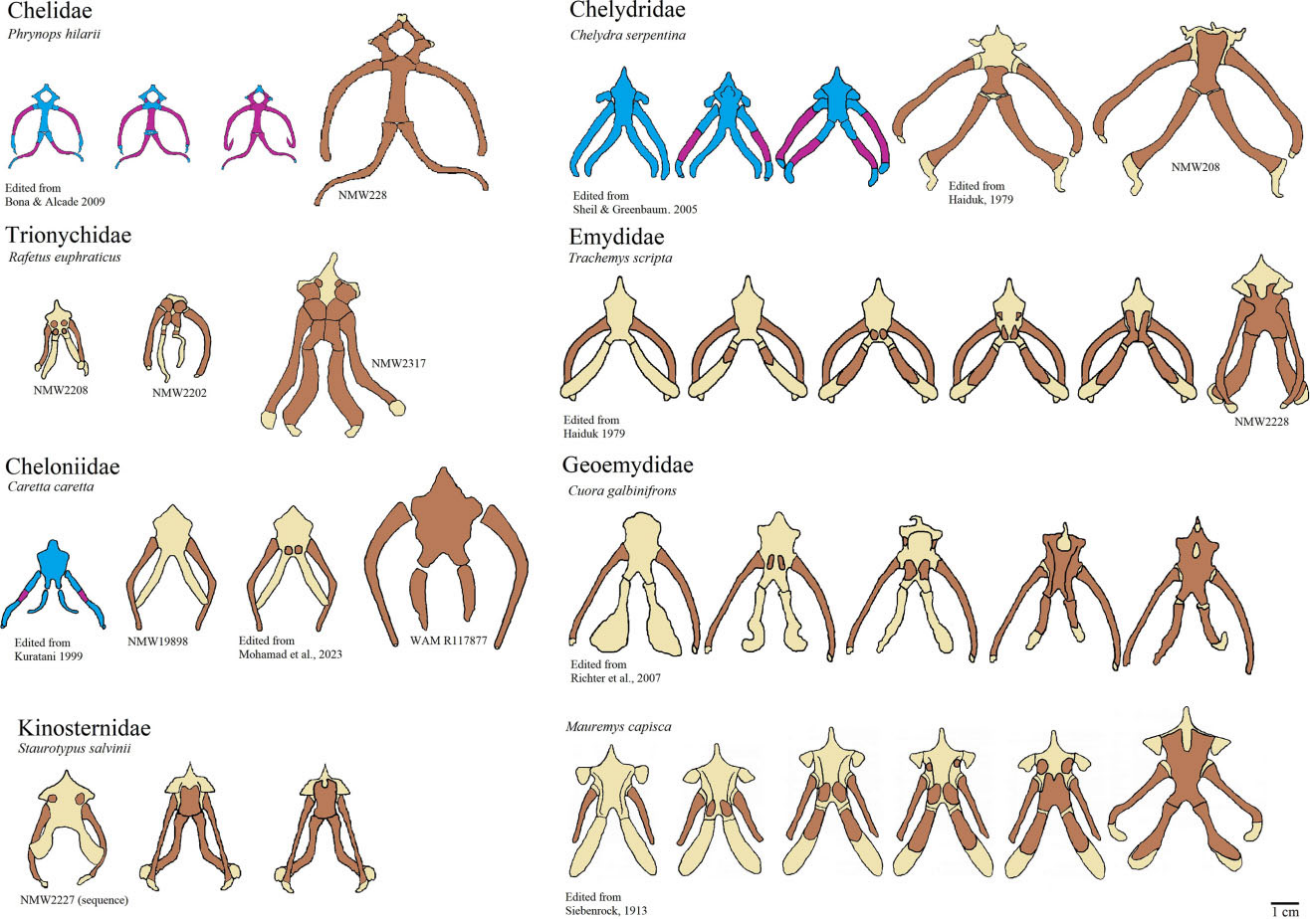
Ontogenetic sequences of ossification in the hyoid apparatus of turtles. Structures colored in blue (cartilage) and red (bone) represent specimens in embryonic stages, while structures in brown (bone) and beige (cartilage) are from post-hatched.

**Fig. 4:**
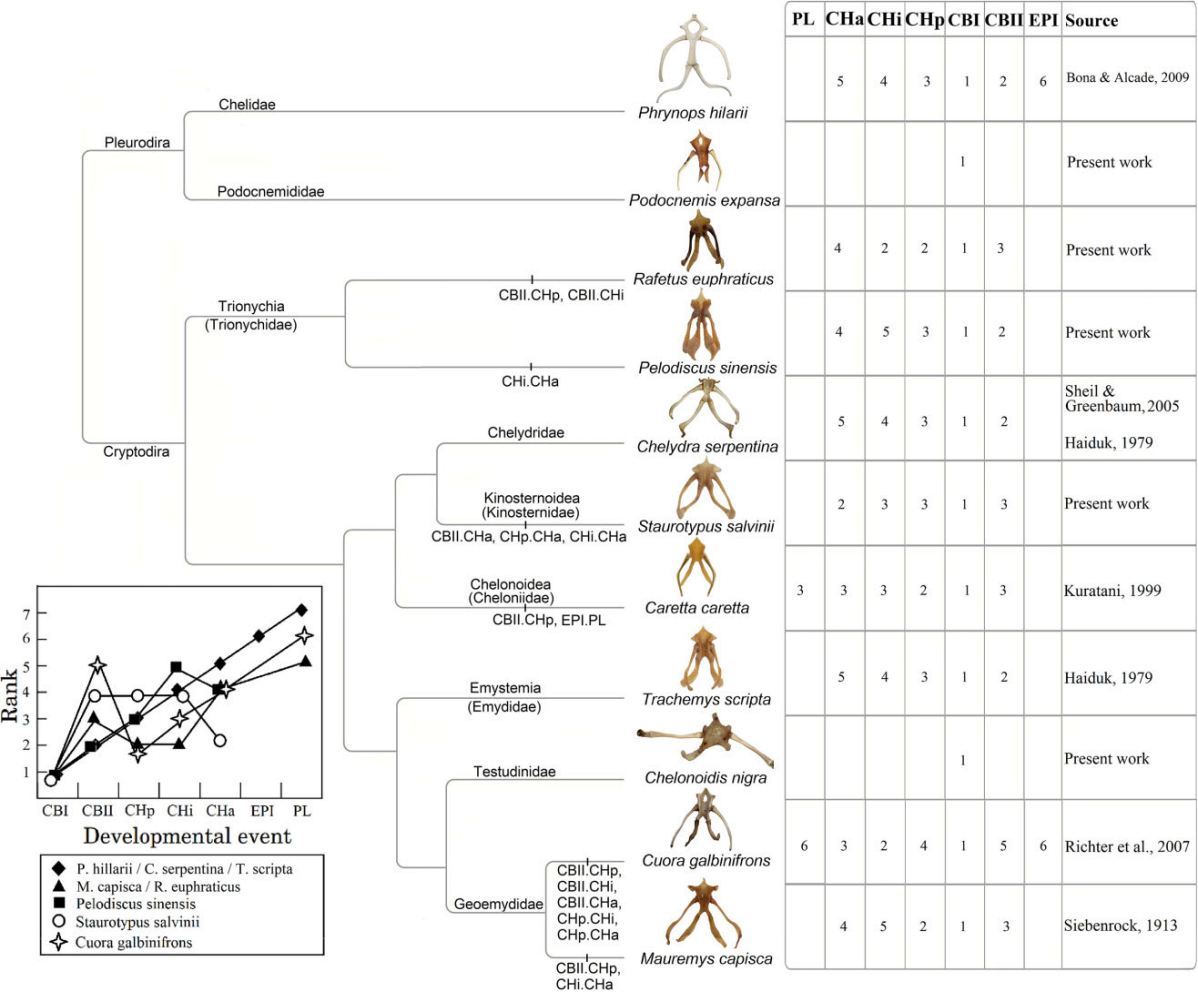
Variation in the ossification sequence of hyoid elements in different turtle species. Heterochronic shifts are listed in the tree as event pairs (Smith 2001). Chelonoidis and Podocnemis are not included in the graphical display of ossification sequence because they only show one ossified structure. CBI, cornus branchialis I; CBII, cornus branchialis II; CHp, posterior portion of the corpus hyoideus; CHi, intermedial portion of the corpus hyoideus; CHa, anterior portion of the corpus hyoideus; EPI, epibranchial I; PL, processus lingualis. Clades are named following Joyce et al. (2021).

**Fig. 5:**
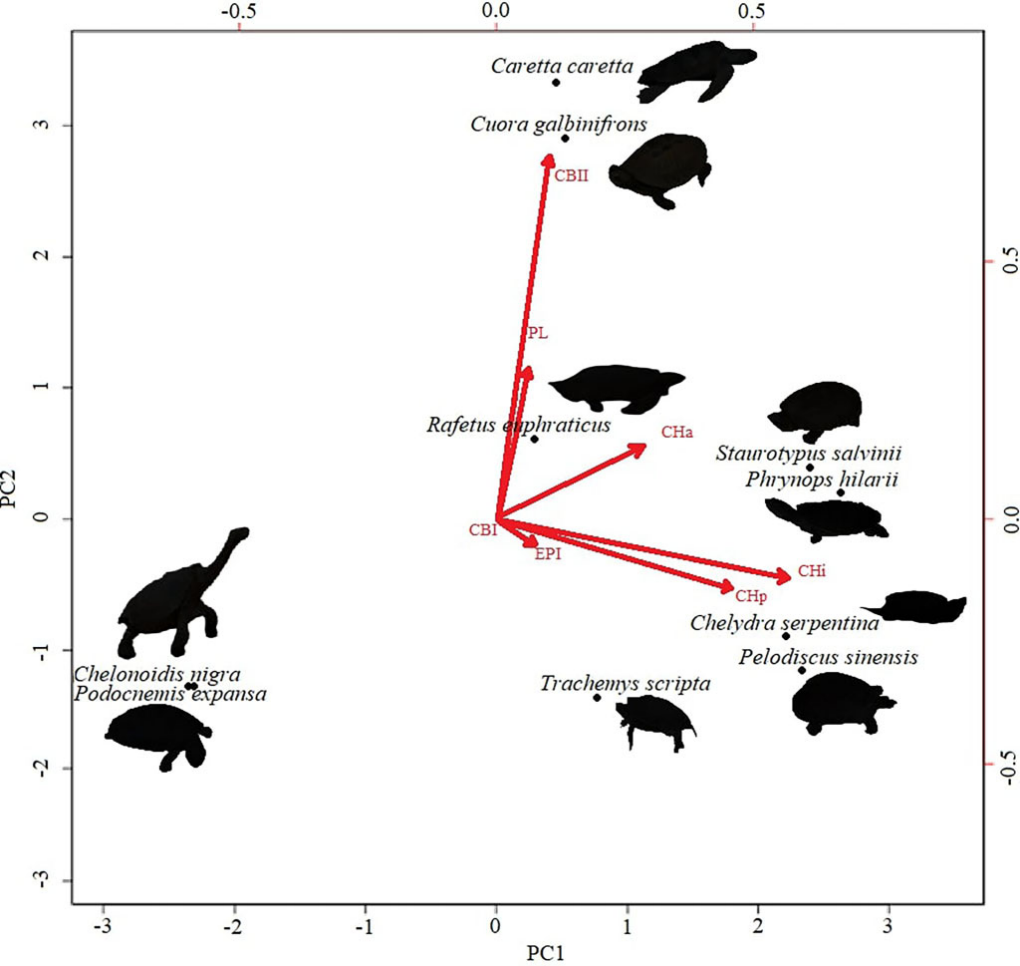
Principal Component Analysis of onset of ossification in the hyoid apparatus of turtles with loadings of single elements. CBI, cornus branchialis I; CBII, cornus branchialis II; CHp, posterior portion of the corpus hyoideus; CHi, intermedial portion of the corpus hyoideus; CHa, anterior portion of the corpus hyoideus; EPI, epibranchial I; PL, processus lingualis.

In total, we observed the onset of ossification from 7 structures of the hyoid apparatus from 10 species belonging to 7 major turtle lineages (“families,” Fig. 3), and conducted analyses based on these data (Figs. 4  and 5).

**Fig. 6:**
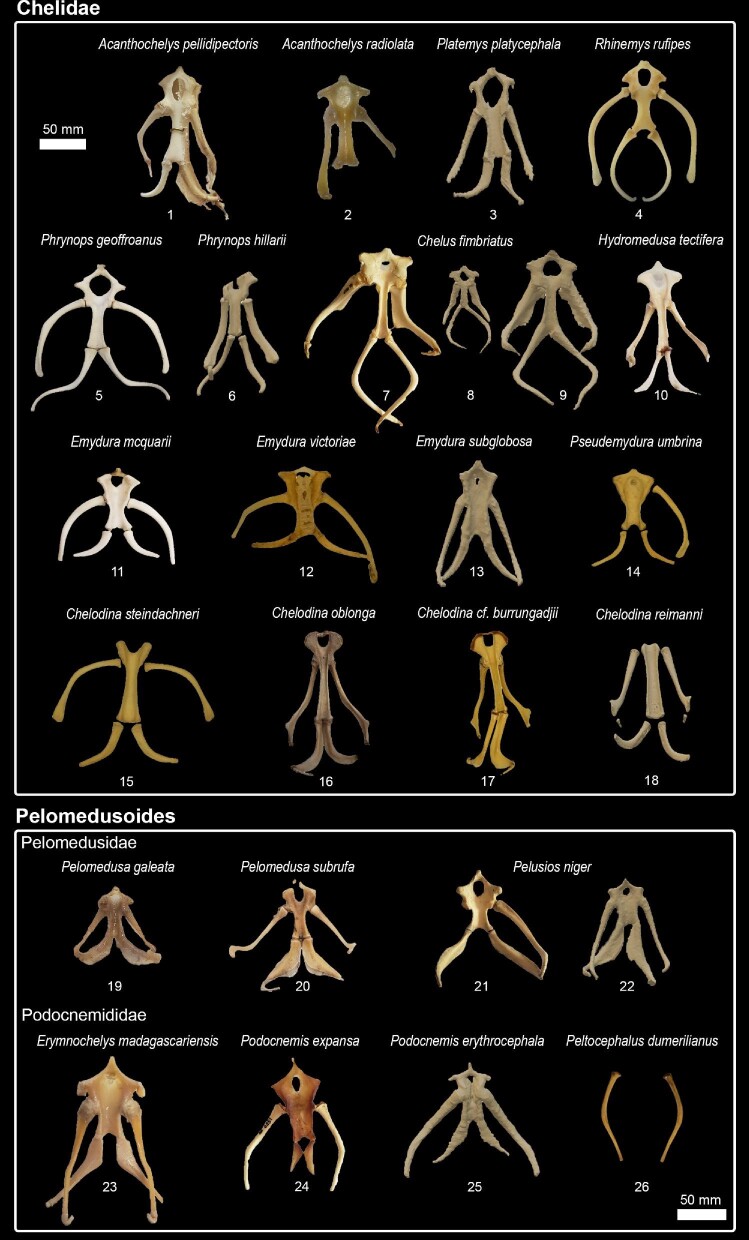
Hyoid apparatus diversity among Pleurodira.

We observed that, in many species, the hyoid apparatus predominantly undergoes ontogenetic changes in their ossification patterns after hatching. The cornu branchiale I is the only structure to ossify before hatching in all four of the species where embryonic hyoid ossification was described (Figs. 2  and 3). It is also always the first—sometimes the only (e.g., tortoises, podocnemidids, and *Carettochelys*)—structure to ossify among all turtle clades. The cornu branchiale II is already ossified in all species besides *Ca. caretta* at hatching time. Only *Ph. hillarii* (Fig. 3), *Chelus fimbriata* (Fig. 6), and *P. Sinensis* (Fig. 2) show some degree of ossification in the corpus hyoideus by the time of hatching, with the hyoid apparatus of *Ph. hillarii* and *Chelu. fimbriata* being nearly completely ossified at this stage (Bona and Alcade 2009; present work, respectively).

We also observed post-hatch ontogenetic changes in seven species, in addition to the four species where embryonic comparison was possible, which present a series of heterochronic shifts in the onset of ossification. The ossification sequence and a list of heterochronic shifts are available in Fig. 4. All studied species present at least one shift in the sequence of post-hatchling ossification, except for *Ph. hillarii, Chely. serpentina* and *T. scripta.* These species do present the same patterns of change; however, this occurs during their earlier embryonic life stages (Fig. 3). The lack of resolution in the sequences of *Ca. caretta, S. salvinii*, and *Cuora galbinifrons* hinder detailed descriptions, but are enough for general comparisons.

In addition to the general pattern of the cornu branchiale I being the first structure to ossify, the epibranchiale I and the processus lingualis are always the last to ossify, in the rare cases where they do (e.g., *Cu. galbinifrons* and *Ca. caretta*). The structure that exerted the greatest influence on distribution of species in the PCA (Fig. 5) was the corpus hyoideus (Cha, Chi, and CHp). The close association of *Cu. galbinifrons* and *Ca. caretta* is due to the fact that these are the only species to show ossification of the processus lingualis. The association of these species is also influenced by the fact that the cornu branchiale II only shows signs of ossification after the corpus hyoideus already started ossifying—a sequence also observed in *R. euphraticus*, but not in *P. sinensis*, a close relative distantly plotted in the PCA. The only groups that do not show any signs of ossification besides of cornu branchiale I are the species belonging to Podocnemididae (*Po. expansa*) and Testudinidae (*Chelo. nigra*) which are, therefore, overlapping in the PCA plot.

**Fig. 7:**
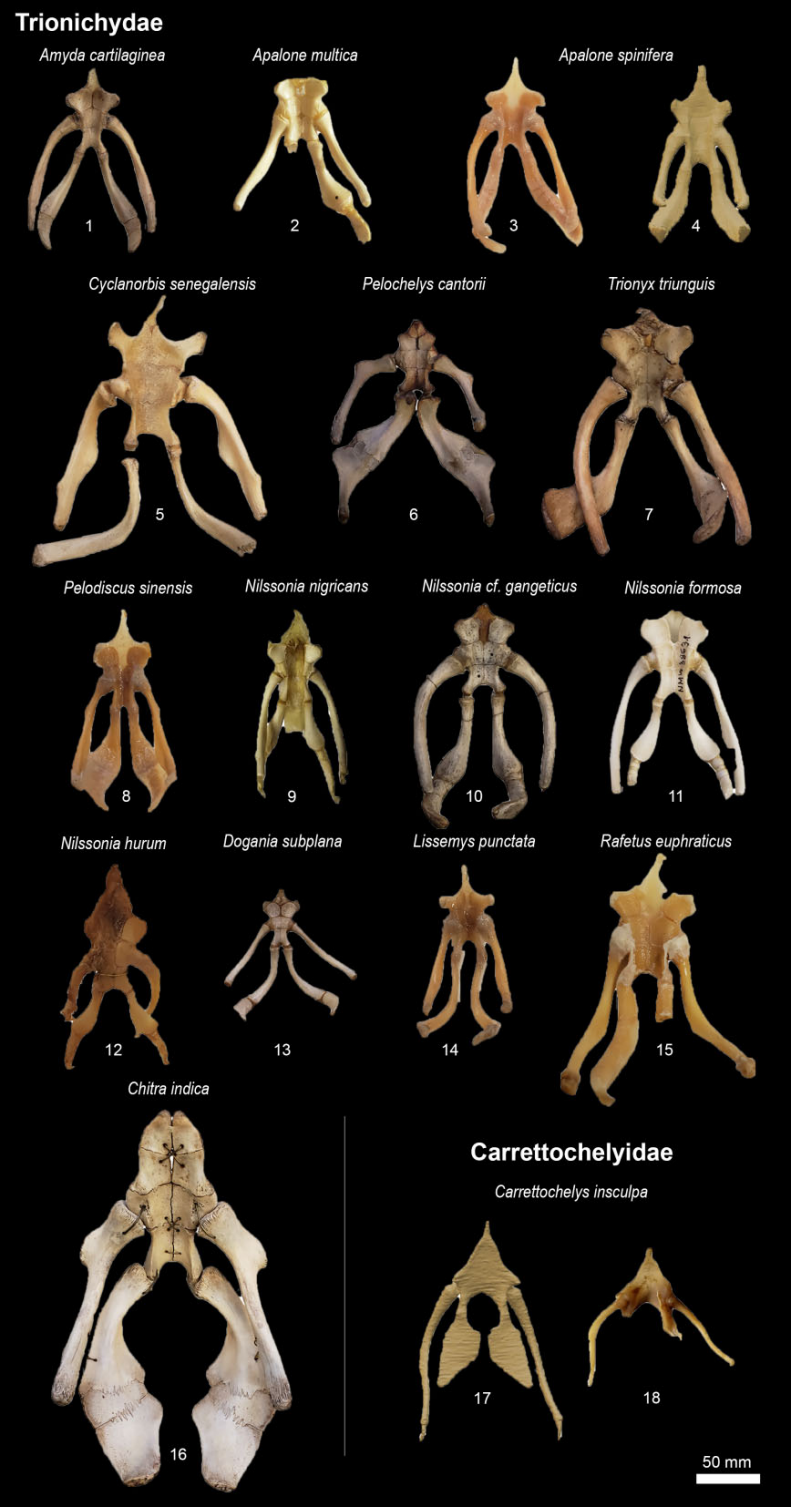
Hyoid apparatus diversity among Trionychia (Cryptodira).

**Fig. 8:**
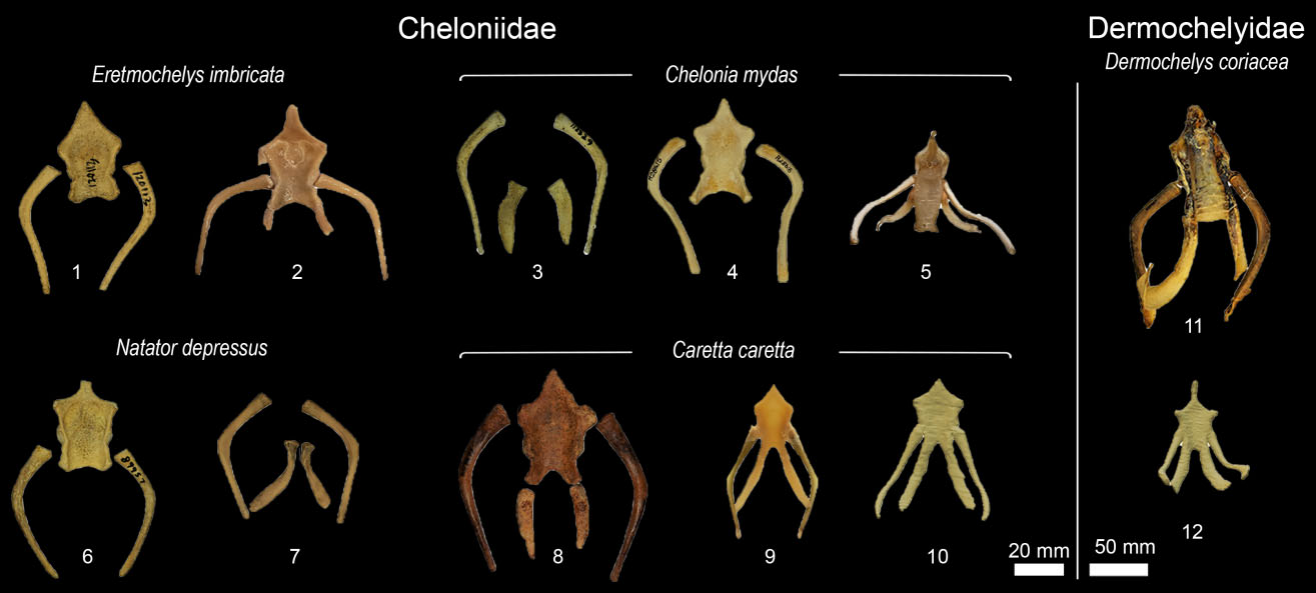
Hyoid apparatus diversity in Chelonioidea (Cryptodira).

**Fig. 9:**
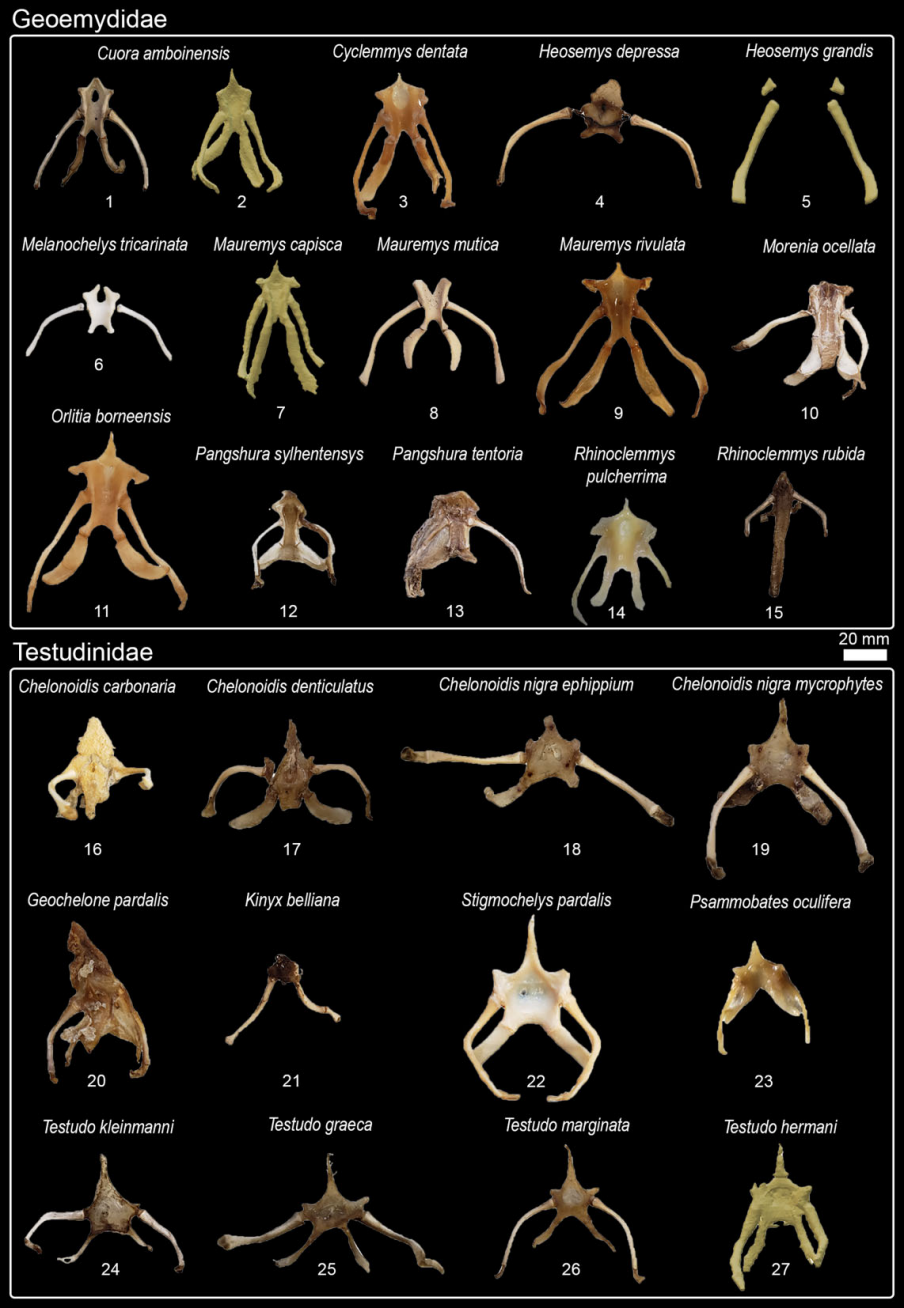
Hyoid apparatus diversity in Testuguria (Cryptodira).

**Fig. 10:**
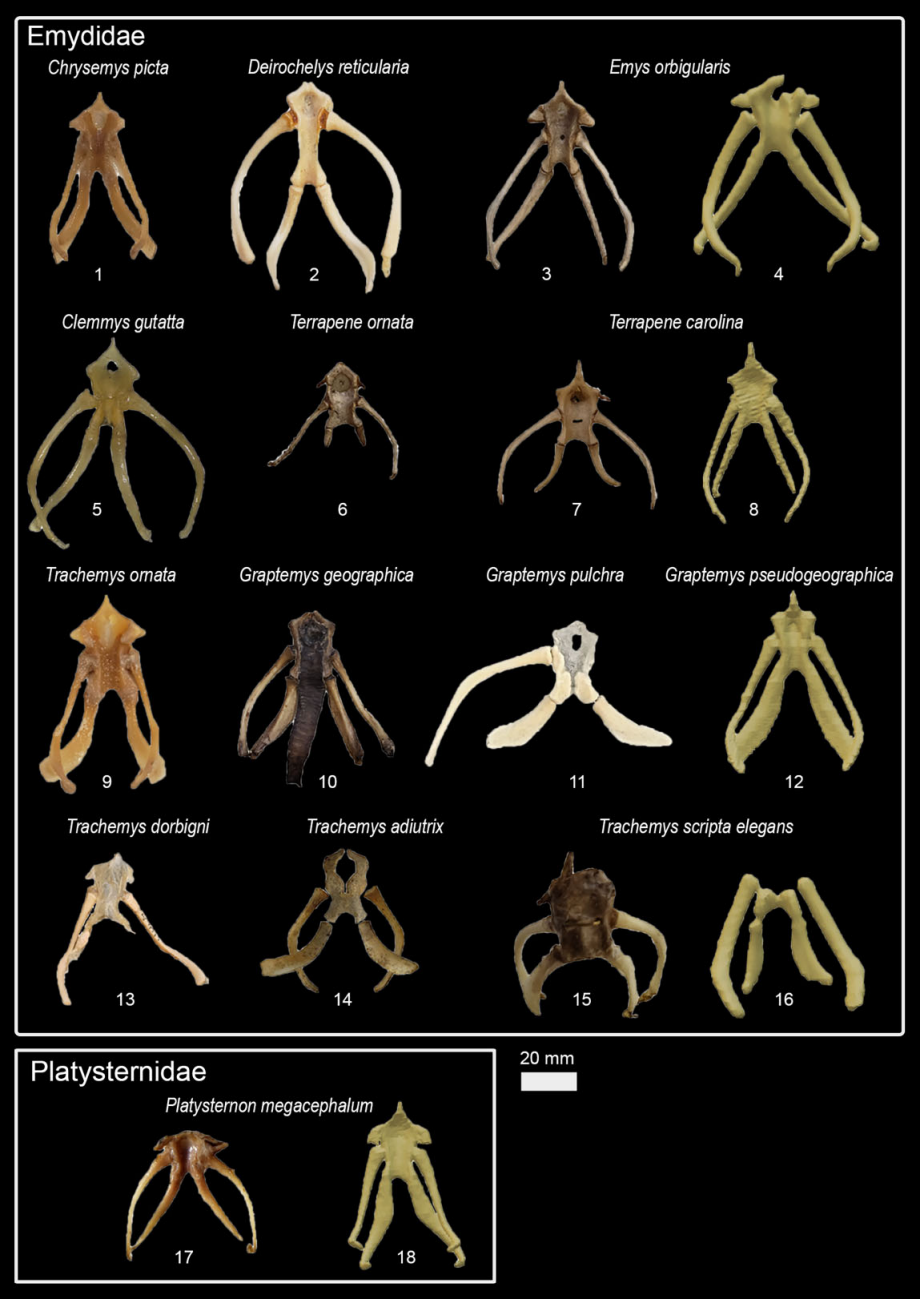
Hyoid apparatus diversity in Emystemia (Cryptodira).

**Fig. 11:**
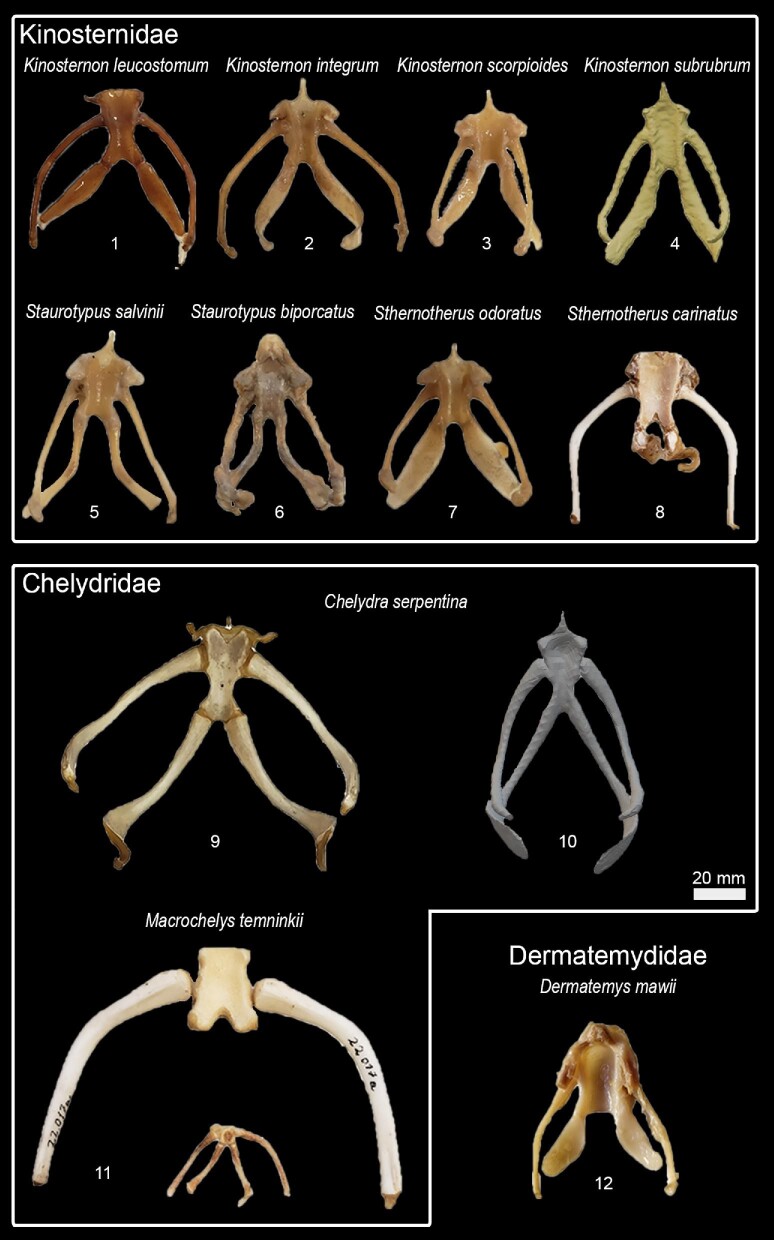
Hyoid apparatus diversity in Chelydroidea (Cryptodira).

**Fig. 12:**
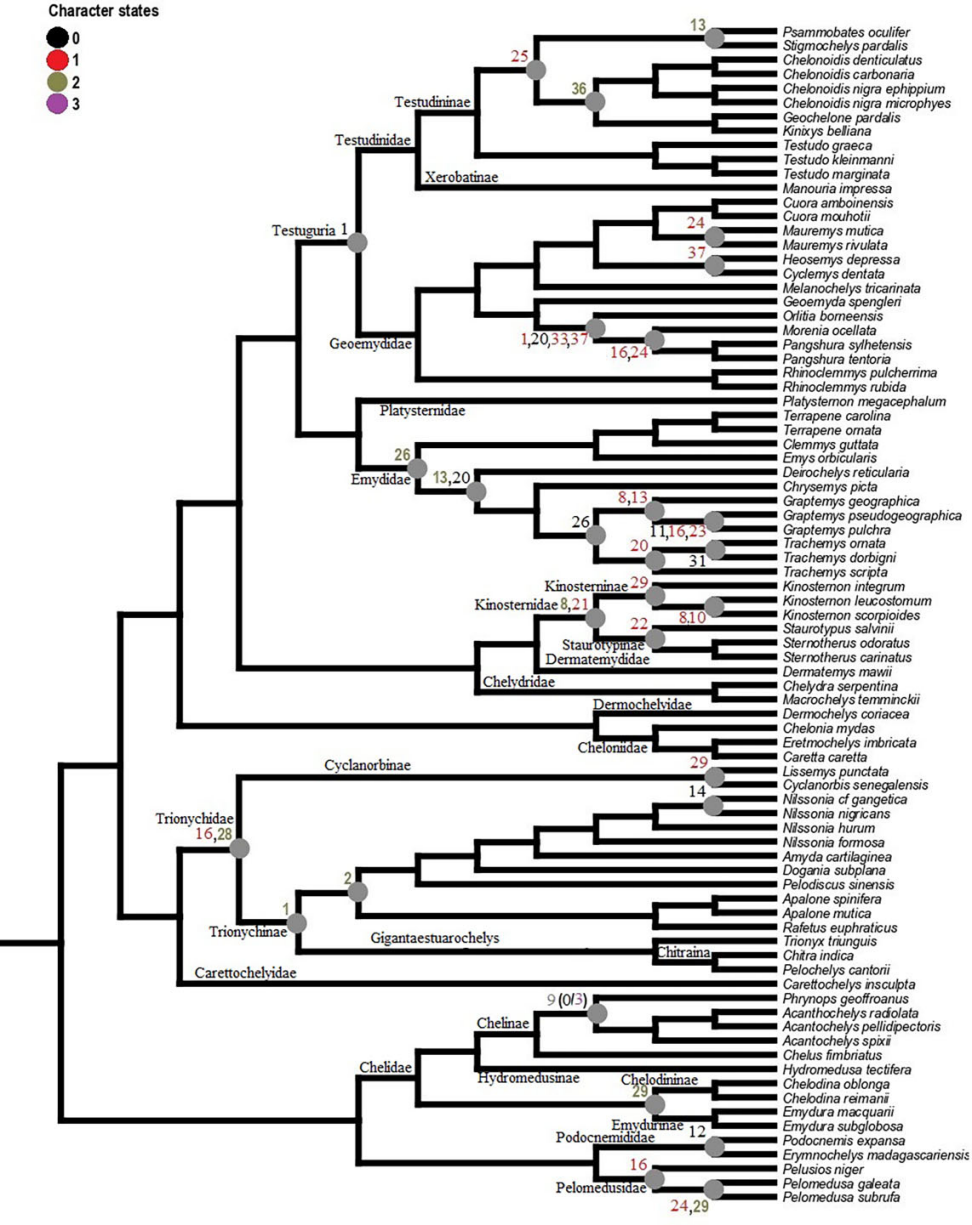
Synapomorphies based on ancestral state reconstruction performed in TNT using the topology of Pereira et al. (2017). Numbers represent characters from Table 3, and colors represent the described character states.

Based on the character matrix (Table 3; Supplementary  Material 1), 41 synapomorphic traits were recovered by TNT (traits can be observed on Figs. 6–11 and the tree on Fig. 12). The absence of the epibranchial II is a synapomorphy of the Testuguria (Testudinidae + Geoemydidae; character 1, state 0; Fig. 9). The structure reappeared in a cartilaginous form in some genera of the Geoemydidae (*Geoemyda* + *Orlitia* + *Morenia* + *Pangshura*). The presence of the epibranchial II as an ossified structure composed by 1 to over 10 round elements describes the Trionychinae (character 1, state 2). The Gigantaestuarochelys clade (*Pelochelys* + *Chitra* + *Trionyx*; Engstrom et al. 2004; Fig. 7) is defined by the presence of only two bony elements in the epibranchial II (character 2, state 1), while the rest of the Trionychinae have several unstandardized elements, often with a different number on each side (character 2, state 2). In Chitraina (*Pelochelys* + *Chitra*; Engstrom et al. 2004), the second pair of epibranchial II forms an edge to which the posterior portion of a cartilaginous structure articulates (character 4, state 1).

The sulculs tracheae separated into four bony elements and the presence of frill in cornu branchiale I are synapomorphies of the Trionychidae (character 28, state 2 and character 16, state 1; respectively). The latter also represents a synapomorphy of the Pelomedusidae and can also be observed in some genera from other lineages such as *Graptemys, Morenia*, and *Pangshura* (Fig. 9).

The presence of attachment hinges on the cornu branchiale I at its medial, curved portion (“elbow's”) defines *Nilssonia* (character 14, state 0; Fig. 7). The sulcus tracheae with a widening of its anterior part is characteristic of *Lissemys* and *Cyclanorbis*, but also of *Kinosternon* (character 20, state 1’ Figs. 7, 11). A widening of the posterior end of the sulcus defines the genus *Pelomedusa* (character 20, state 2; Fig. 6).

Epibranchials I shaped like a hook (character 12, state 0) may define Podocnemididae, but there is a need for checking the state of this character in *Peltocephalus* (Fig. 6).

The genus *Pelomedusa* can be defined by the opening of the anterior portion of the fenestra hyoidea (character 24, state 1; Fig. 6).

Kinosternidae is defined by the isosceles shape of the processus lingualis (character 21, state 1) and the continuous width of the cornu branchiale II (character 8, state 2; Fig. 11). Within this clade, Kinosterninae lacks the fenestra hyoidea, while it is present in Staurotypinae (character 22). The presence of a frill in cornu branchiale II is characteristic of a clade within *Kinosternon*, ecompassig *K. leucostomum* and *K. scorpioides* (character 10, state 1).

The fenestra hyoidea as wide as the space between the processi lateralis intermedius is characteristic of the Emydidae (character 26, state 2; Fig. 10). The fenestra hyoidea subdivided into two separate openings defines the Testudininae except for *Testudo* (character 25, state 1; Fig. 9).

The distribution of the referred synapomorphic traits can be visualized along the turtle phylogenetic tree (Fig. 12).

## Discussion

The hyoid apparatus underwent recurrent morphological changes during the evolutionary history of turtles, including shape, relative size, amount of ossification, and the sequence of ossification. Previous studies have shown that different turtle taxa present distinct amounts of ossified structures in their hyoid apparatus (e.g., Haiduk 1979; Richter et al. 2007) and that the degree of ossification of the hyoid apparatus is subjected to ontogenetic changes, with progressive ossification associated with age (Tanner and Avery 1982). The degree of ossification of the hyoid apparatus, along with several other morphological traits have also been shown to strongly correlate with feeding behavior—with three main feeding modes within Testudines (Lemell et al. 2019).

## Hyoid morphology and feeding modes

Most turtles feed through jaw prehension, a trait thought to be plesiomorphic and present in early testudinatan lineages such as the possibly semiterrestrial *Proganochelys quenstedtii* (Lemell et al. 2019). This feeding method allows food capture both in water and land and is associated with a stout and highly ossified corpus hyoideus as well as a large processus lingualis. These traits grant some degree of water suction, enough to counterbalance the impact caused by biting and head projection while capturing food underwater (Natchev et al. 2015), but also allow food manipulation by the tongue—which is supported by the processus lingualis.

Some non-directly related turtle species are specialized suction feeders (i.e., food intake occurs through a high suction force caused by a negative mouth pressure, resultant from a swift depression of the hyoid apparatus), and present several convergent morphological traits. One of the most obvious characteristics is the strong elongation of several substructures of the hyoid apparatus, making it proportionally much larger compared with non-suction feeders. The corni branchiali and the corpus hyoideus are among some of the elongated substructures. The hyoid apparatus of suction feeders also presents extreme degrees of ossification—a trait that can be observed in suction feeders from other aquatic tetrapods such as whales and pinnipeds (Wainwright et al. 2015)—including rarely ossified structures such as the epibranchials I. The need for stability and strength to resist the force, associated with sudden movements mediated by developed musculature, required in suction behavior may explain the high degrees of ossification. In contrast to species that use jaw prehension for food intake—which show ontogenetic changes in the degree of ossification of the hyoid apparatus—suction feeders exhibit near complete ossification during embryologic development (e.g., *Ph. hillarii* and *Chelu. fimbriata*; Bona and Alcade 2009; present work, respectively). Although caution in interpretations based on temporal shifts is advisable as they do not intrinsically carry biological significance (Werneburg et al. 2009, 2015b), the rapid ossification in suction feeders may be associated with the need for hatchlings to engage in suction behavior as early as a week after hatching.

Suction-feeding turtles tend to have flattened skulls, reduced jaws, and elongated necks (e.g., *Chelu. fimbriata* snake-necked turtles from the genus *Chelodina*, some softshelled turtles such as *Chitra* and *Cyclanorbis*, and the sole suction feeder within emydids: *Deirochelys reticularia*; Bramble and Wake 1985; Lemell et al. 2002; Whishaw and Karl 2019; Hermanson et al. 2022). In addition, they tend to develop broad attachment sites for musculature in the cornu brachiale I (e.g., *Cyclanorbis, Pelochelys, Chitra, Chelus, and Chelodina*). Similar trends can be observed in association to the diversity of the musculature in the head of turtles, and in its integration with the hyoid apparatus (Werneburg 2011; Yoshida et al. 2023, respectively).

The third type of feeding has only been reported for tortoises and is based on lingual prehension (Lemell et al. 2019). A well-developed adhesive tongue is protruded and captures the food, similarly observed in many amphibians, including several lineages of anurans and salamanders (Barrionuevo 2016), and more extremely in chameleons (de Groot and van Leeuwen 2004). The hyoid apparatus of tortoises is mostly cartilaginous and relatively short. It has been hypothesized that there is an inverse correlation between the sizes of the tongue and the hyoid apparatus and that animals that feed on land have minor degrees of ossification, as it requires more skeletal flexibility when chewing and moving the tongue (Lemell et al. 2000). Although the latter has been proven inaccurate by the presence of cartilaginous hyoid apparatus in aquatic species and vice-versa, we also observed the trend described by the first hypothesis. Furthermore, we observed a strong inverse correlation between the size of the hyoid apparatus and the processus lingualis—statistical tests are required to confirm this. While most turtles only use their tongues for food manipulation, tortoises present a comparatively high elongation of the processus lingualis associated with their feeding mode and the need for tongue extension (Natchev et al. 2015).

An especially enlarged processus lingualis can also be observed in *M. temminckii* (Gregory 1946), most probably associated with the tongue movements performed by the species to deceive fish prey. Conversely, suction feeders have shrunken—sometimes absent—processus lingualis and tongue (e.g., some chelids, trionychids, and *D. reticularia*). The tongue needs to be reduced not to interfere with the suction, resulting in the reduction of the processus lingualis—a trait also observed in suction-feeding frogs (e.g., Pipidae; Barrionuevo 2016). In trionychids, the only clade where the corpus hyoideus ossifies in subdivisions of three bone pairs (Ch. 28[2]), specialized suction feeders developed an extra pair of bones in the anterior portion of the corpus (e.g., *Pelochelys* and *Chitra*). This extra pair of ossifications substitutes the broad anchoring site of the processus lingualis present in other non-suction feeding species of the clade.

## Hyoid morphology and sound production

The large processus lingualis and tongue protrusion in tortoises has also been associated with sound production (Sacchi et al. 2004). Theoretically, tortoises would only be able to produce open-mouth vocalizations as it requires the full extension of the proximal portion of the larynx, which happens through the descent of the hyoid apparatus and the protrusion of the tongue. The cartilaginous nature of the hyoid apparatus of tortoises is thought to be associated with the need for lingual flexibility (Wochesländer et al. 1999), essential in behaviors such as feeding and sound production. Nevertheless, tortoises also do produce closed-mouth vocalizations and most turtles, in fact, seem to produce mostly closed-mouth vocalizations (GJC, personal observation). The movements are then performed by the hyoid apparatus and can be observed in animals interacting both under water and on land (GJC, personal observation). This ability to produce closed-mouth vocalizations is also widespread among birds and crocodilians (Riede et al. 2016), likely the closest living relatives of turtles.

Furthermore, the correlation between the presence of cartilaginous corpus hyoideus and a large flexible tongue is not always simple—and neither can it be explained by feeding habits, habitat, or vocal behavior. Although, like tortoises, some terrestrial taxa such as *Rhinoclemmys, Cuora*, and *Terrapene* produce sounds (Jorgewich-Cohen et al. 2022) and have large tongues (Natchev et al. 2009), they show high degrees of ossification in the hyoid apparatus. However, highly aquatic and vocal species such as *Po. expansa* and *Carettochelys insculpta* (Ferrara et al. 2014, 2017) have a cartilaginous hyoid apparatus with comparatively small tongues (Silveira et al. 2015). Some suction feeders are also known to produce sounds, such as *Chelodina* and *Chelus* (Giles et al. 2009; Jorgewich-Cohen et al. 2022).

The mechanisms of sound production have not been investigated in any turtle species apart from *Testudo*. Göppert (1937) observed that turtles lack vocal cords, and Siebenrock (1898) described the larynx of both turtles and crocodilians to be supported by the corpus hyoideus—the larynx of turtles being the most morphologically diverse among reptiles (Siebenrock 1899). Considering that many turtle species do not present vocal folds—except for some lineages such as trionychids (Göppert 1937; Ogushi 1913), and that the mechanisms of sound production are “randomly” distributed among reptiles with several evolutionary shifts (Sacchi et al. 2004; Russel and Bauer 2021), determining the main structure responsible for sound production among all turtle clades is currently a hopeless task. Our finding that two major lineages of Testudinidae can be defined by the presence of fused (*Manouria* and *Testudo*, and probably *Gopherus*) and doubled (all Testudininae except for *Testudo*) fenestra hyoidea brings little insights regarding sound production, especially because nearly all species within Testudinidae have been shown to produce sounds (Ferrara et al. 2013; Jorgewich-Cohen et al. 2022). After Sacchi et al. (2004), the only couple of studies to correlate morphological structures to acoustic behavior were focused on sea turtles (Fraher et al. 2010; Oliveira et al. 2023). In both cases, vocalizations were only marginally discussed and no strong correlations were established—only suggesting that glottal closure mechanisms may be related to sound production (Oliveira et al. 2023).

Considering the high number of morphological shifts to which the hyoid apparatus of turtles was subjected, inferring general links between morphological traits and specific ecological/behavioral trends can be difficult. However, studies focused on musculature embedding may be helpful to clarify some of these correlations. The same applies to the use of such morphological characters in taxonomic and for phylogenetic inferences, which should consider the high number of homoplasies.

## Phylogenetic and taxonomic inferences

Few studies have used traits of the hyoid apparatus of turtles in phylogenetic inferences (e.g., Hirayama 1985; Meylan 1987; Gaffney and Meylan 1988; Yasukawa et al. 2001). Among these, some characters—such as the ossification of cornu branchiale II (Hirayama 1985; Yasukawa et al. 2001), interpreted as a synapomorphy that describes a group composed of tortoises and some geoemydids—were later refuted (Joyce and Bell 2004).

The analysis of sequence heterochrony may suffer from biases due to pseudo shifts, which appear because of different resolutions and weightings of ontogenetic series (Jeffery et al. 2005; Werneburg and Sánchez-Villagra 2009). Siebenrock (1913) described the ossification series of *M. caspica* and *Testudo graeca*, the first being included in the present work. Although in this report he described the ossification sequence in the hyoid of *T. graeca*, no specimen of Testudinidae observed by us shows any signs of ossification beyond the cornu branchiale I—a trait also reported by other authors (Natchev et al. 2015). Although unlikely, his descriptions may result from misidentification or animals with a special condition (i.e., malformation). Another alternative is that he described regional traits. We observed different patterns in different populations in certain lineages, which may represent some degree of plasticity. For example, the corpus hyoideus of adult *Ca. caretta* from Western Australia are highly ossified. We did not find a single specimen from other populations with the same trait—a topic to be investigated further in future research. Nevertheless, this difference is rather minor when compared with the difference we documented in *T. graeca*. As we cannot find a clear explanation for the differences, we decided to exclude the observation from Siebenrock on this species from our analysis as it also does not fit the overall and otherwise convincing pattern described herein.

The complete absence of ossification beyond the cornu branchiale I in tortoises has been previously associated with the fact that this is the only intrinsically terrestrial taxon within Testudines (Siebenrock 1898; Lemell et al. 2000). However, previous studies lack samples from highly aquatic lineages that show similar ossification patterns to the ones found in tortoises. For example, in Podocnemididae, and possibly Carettochelyidae (needs confirmation through the observation of ontogenetically advanced specimens), there are no ontogenetic changes in the ossification patterns of the hyoid apparatus, with cornu branchiale I being the only structure to ossify—which happens during embryonic development (Vieira et al. 2010). Contrarily, terrestrial species that do not belong to Testudinidae, such as *Terrapene* and *Cuora*, show a high degree of ossification (Summers et al. 1998; Richter et al. 2007; present work).

Regardless, some of the character states observed in the present work represent synapomorphies that describe specific turtle lineages and are, therefore, useful taxonomic traits. The Trionychinae can be defined based on the presence of 1 to over 10 round bony elements that compose the epibranchial II (charcter 2). This morphology is not present in species of the sister taxon, Cyclanorbinae (although our dataset lacks samples of *Cycloderma*, the structure is absent in samples from *Cyclanorbis* and *Lissemys* we assessed). The number of bony elements that compose the epibranchial II has suffered many changes during the evolutionary history of Trionychinae—sometimes not even being symmetrical in the same specimen—but seems to be stable within the clade Gigantaestuarochelys (*Pelochelys* + *Chitra* + *Trionyx*; Engstrom et al. 2004), that possess always two, which are partially fused in *Trionyx*. In Chitraina (*Pelochelys* + *Chitra*; Engstrom et al. 2004), the second pair of epibranchial II forms an edge to which the posterior portion of a cartilaginous structure articulates. The anterior part of the cartilage contacts an edge formed by the posterior portion of the cornu branchiale II, passing over the first ossified structure of the epibranchial II (character 4[1], Fig. 7, specimens 6 and 16). The edge formed by the cornu branchiale II is also present in *Trionyx* (Fig. 7, specimens 7), constituting a synapomorphy of Gigantaestuarochelys, while the cartilaginous structure only describes Chitraina.

**Fig. 13:**
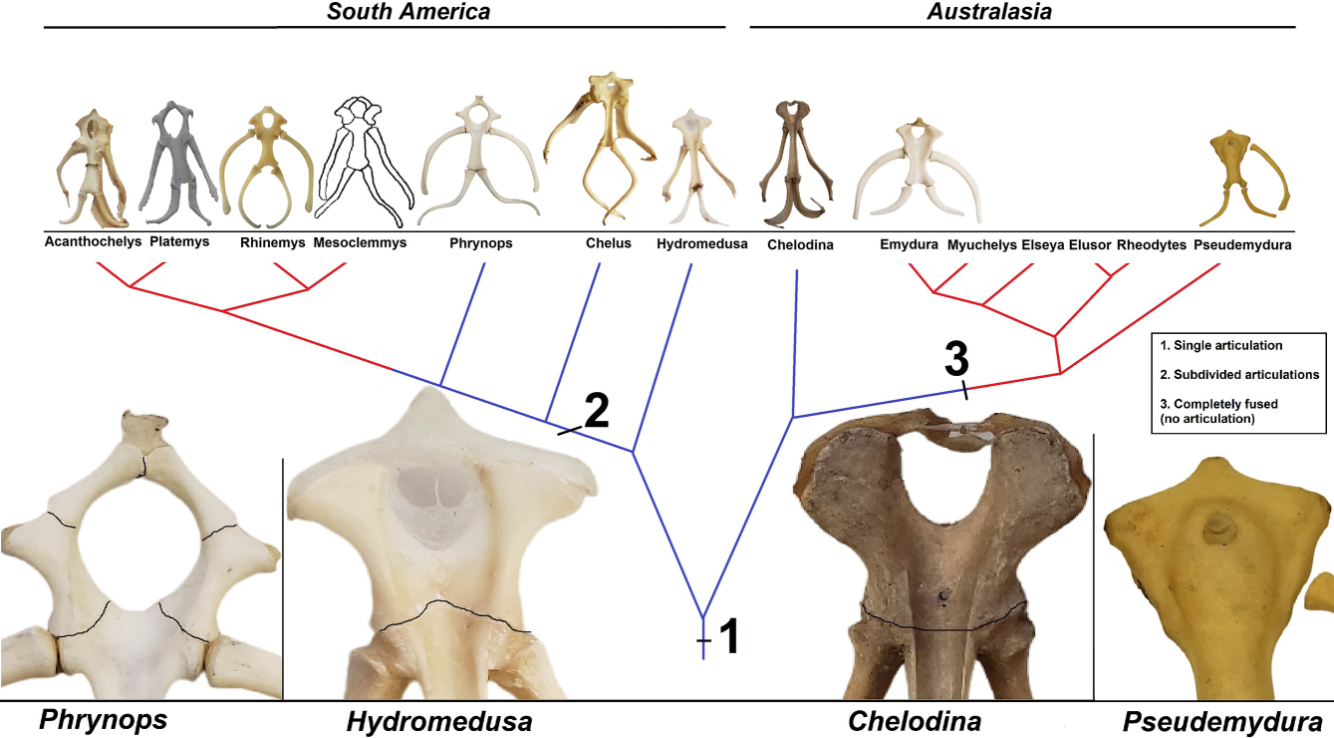
Corpus hyoideus sutures in Chelidae. Blue lines represent suction feeding and red line represents jaw prehension. Information about the genus Mesoclemmys was extracted from Zangerl and Medem (1958).

The only other lineages of turtles in which the epibranchial II ossifies are the pleurodirans *Chelodina* and *Hydromedusa*. In these cases, it is a single structure with a rod-like shape, much smaller and thinner than the structures observed in Trionychinae. Furthermore, both *Chelodina* and *Hydromedusa* are the only lineages in which the posterior part of the corpus hyoideus that constitute the sulcus tracheae and the anterior part are separated by a suture (Fig. 13). These lineages could have retained the ancestral character state within Chelidae, as they are respectively the sister taxa to all Australasian and South American species. The corpus hyoideus is completely fused within the Australian chelids (Emydurinae + Pseudemydurinae; sensu Joyce et al. 2021), and is partitioned into five separate bones in Chelinae (sensu Joyce et al. 2021). The differences in the hyoid apparatus within Chelidae support the hypothesis of reciprocal monophyly of the South American and the Australasian clades, and the convergence hypothesis of neck elongation (Seddon et al. 1997; Shaffer et al. 1997; Georges et al. 1999; Fujita et al. 2004; Sánchez-Villagra et al. 2007; Ferreira et al. 2018). Nevertheless, the degree of plesiomorphies make it hard to infer ancestral states, especially considering that the outgroup—i.e., Pelomedusidae and Podocnemididae—show completely fused and ossified, and cartilaginous corpus, respectively.

Subdivisions of the corpus hyoideus are also present in trionychids, which show a series of paired bones, most frequently constituted by three, but sometimes by four pairs (Ch. 28 [2], partially). This is a synapomorphy that can be used to describe pan-trionychids, as it is observable in specimens preserved in the fossil record, such as *Palaeoamyda messeliana* (Cadena 2016). Unfortunately, most fossil turtles do not preserve the hyoid apparatus (e.g., Cadena and Parham 2015; Shao et al. 2018; Joyce et al. 2021; Li et al. 2022), most often only conserving the cornu branchiale I, as it is the only structure to always ossify. Nevertheless, the present study sheds light on the evolutionary patterns of turtle hyoid apparatus morphology that can be helpful to recognize both ecological features and taxonomic affiliation in extinct and living species.

## Supplementary Material

obae014_Supplemental_Files
