# Supplementary material for: Morphological Diversity of Turtle Hyoid Apparatus is Linked to Feeding Behavior
Source: Integr Org Biol. 2024 May 2;6(1):obae014. doi: 10.1093/iob/obae014 (PMC11090499; doi:10.1093/iob/obae014)
Supplement: obae014_Supplemental_Files [file obae014_supplemental_files.zip › Supplemental Material 2 ancestral_state_recs-ambiguities.zip!/ch18.pdf]

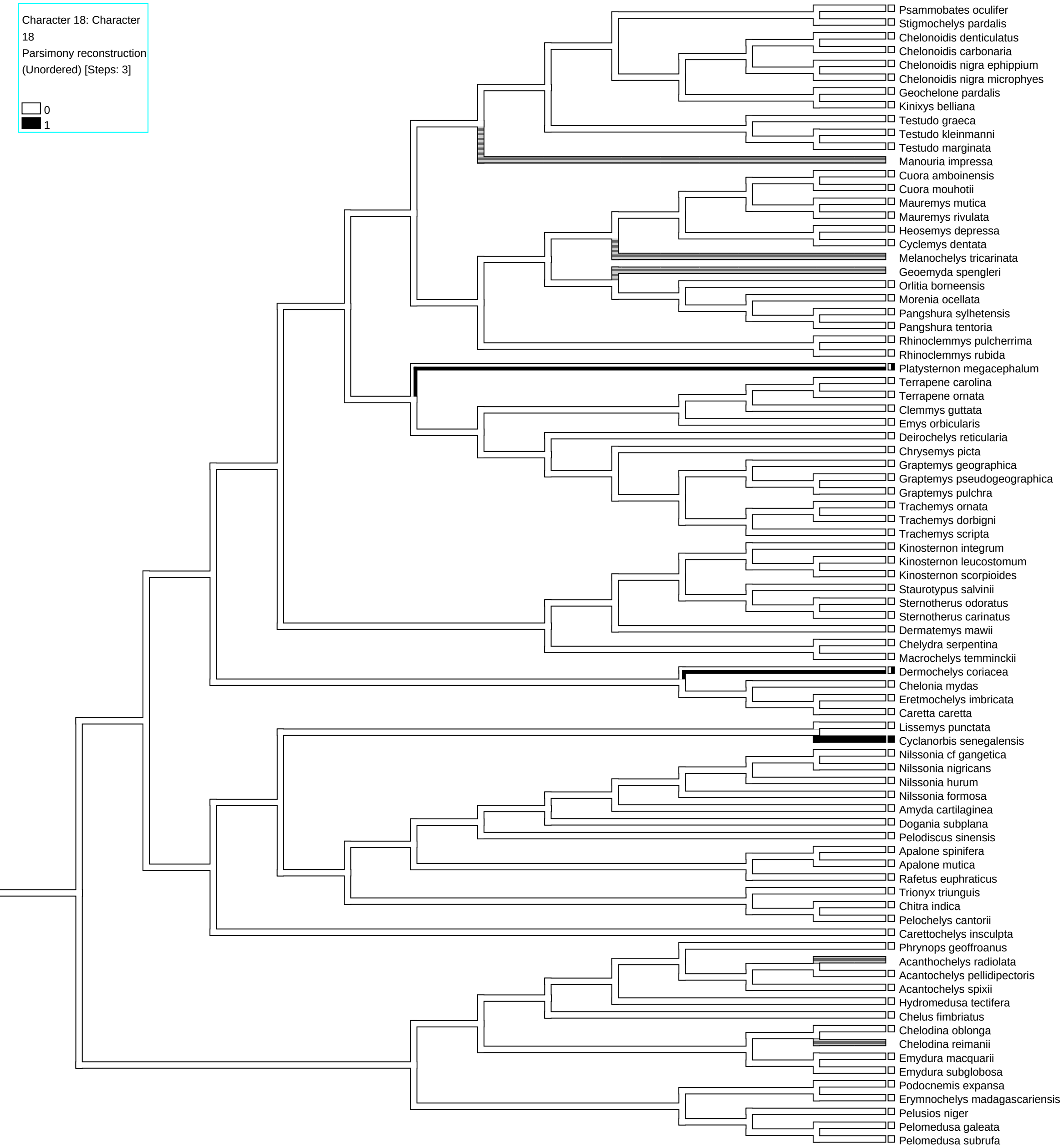
Character 18: Character 18
Parsimony reconstruction (Unordered) [Steps: 3]
0
1
Psammobates oculifer
Stigmochelys pardalis
Chelonoidis denticulatus
Chelonoidis carbonaria
Chelonoidis nigra ephippium
Chelonoidis nigra microphyes
Geochelone pardalis
Kinixys belliana
Testudo graeca
Testudo kleinmanni
Testudo marginata
Manouria impressa
Cuora amboinensis
Cuora mouhotii
Mauremys mutica
Mauremys rivulata
Heosemys depressa
Cyclemys dentata
Melanochelys tricarinata
Geoemyda spengleri
Orlitia borneensis
Morenia ocellata
Pangshura sylhetensis
Pangshura tentoria
Rhinoclemmys pulcherrima
Rhinoclemmys rubida
Platysternon megacephalum
Terrapene carolina
Terrapene ornata
Clemmys guttata
Emys orbicularis
Deirochelys reticularia
Chrysemys picta
Graptemys geographica
Graptemys pseudogeographica
Graptemys pulchra
Trachemys ornata
Trachemys dorbigni
Trachemys scripta
Kinosternon integrum
Kinosternon leucostomum
Kinosternon scorpioides
Staurotypus salvinii
Sternotherus odoratus
Sternotherus carinatus
Dermatemys mawii
Chelydra serpentina
Macrochelys temminckii
Dermochelys coriacea
Chelonia mydas
Eretmochelys imbricata
Caretta caretta
Lissemys punctata
Cyclanorbis senegalensis
Nilssonia cf gangetica
Nilssonia nigricans
Nilssonia hurum
Nilssonia formosa
Amyda cartilaginea
Dogania subplana
Pelodiscus sinensis
Apalone spinifera
Apalone mutica
Rafetus euphraticus
Trionyx triunguis
Chitra indica
Pelochelys cantorii
Carettochelys insculpta
Phrynops geoffroanus
Acanthochelys radiolata
Acantochelys pellidipectoris
Acantochelys spixii
Hydromedusa tectifera
Chelus fimbriatus
Chelodina oblonga
Chelodina reimanii
Emydura macquarii
Emydura subglobosa
Podocnemis expansa
Erymnochelys madagascariensis
Pelusios niger
Pelomedusa galeata
Pelomedusa subrufa
